# Postnatal mental distress in relation to the sociocultural practices of childbirth: An exploratory qualitative study from Ethiopia^☆^

**DOI:** 10.1016/j.socscimed.2009.07.043

**Published:** 2009-10

**Authors:** Charlotte Hanlon, Rob Whitley, Dawit Wondimagegn, Atalay Alem, Martin Prince

**Affiliations:** a King's College London, Institute of Psychiatry, Health Services and Population Research Department, De Crespigny Park, Camberwell, London SE5 8AF, UK; b Dartmouth Psychiatric Research Center, Dartmouth Medical School, Lebanon, NH, USA; c Department of Psychiatry, Faculty of Medicine, Addis Ababa University, Addis Ababa, Ethiopia

**Keywords:** Ethiopia, Sub-Saharan Africa, Postnatal depression, Childbirth, Gender, Postnatal period

## Abstract

Sociocultural patterning of the postnatal period in non-Western settings has been hypothesised to protect against postnatal depression. In 2004, in a predominantly rural area of Ethiopia, we conducted 25 in-depth interviews and five focus group discussions with purposively selected participants including perinatal women, fathers, grandmothers, traditional and religious leaders, birth attendants and community leaders. Our main objectives were (1) to examine societal recognition of problematic distress states in the postnatal period and relate this to Western conceptualisations of postnatal depression and (2) to relate the occurrence of distress states to sociocultural patterning of the postnatal period. Inductive analysis was employed to identify salient themes. Participants spontaneously described culturally problematic distress states occurring in the postnatal period, although did not consider them to be illness. Vulnerability and danger of the postnatal period was emphasised, with risk of supernatural attack and physical harm leading to distress states. Participants also spoke of how gender disadvantage and economic strain intersect with cultural patterning of the postnatal period, threatening mental health due to the resulting disappointed expectations and exclusion, as well as exacerbation of pre-existing problems. Cultural dissonance, where a person's beliefs or actions are out of kilter with strong prevailing cultural norms, may be an important risk factor for postnatal distress in rural Ethiopia, where the postnatal period is extensively culturally elaborated.

## Introduction

Postnatal depression complicates the puerperium for an estimated 13% of women in Western countries (O'Hara & Swain, 1996). In contrast, an influential review of anthropological accounts from non-Western societies found little evidence for postnatal depression (Stern & Kruckman, 1983). The authors hypothesised that, across non-Western cultures, common elements of sociocultural structuring of the postnatal period might protect against development of postnatal depression: ‘(1) Cultural patterning of a distinct post-partum period, (2) protective measures designed to reflect the vulnerability of the new mother, (3) social seclusion, (4) mandated rest, (5) assistance in tasks from relatives and/or midwife, and (6) social recognition of new social status through rituals, gifts or other means’ (Stern & Kruckman, 1983). In Western cultures, the rise of modern obstetric practices has been argued to alienate women from these important components of the rite of passage into motherhood, rendering them susceptible to postnatal mental disorder (Davis-Floyd, 1987; Seel, 1986).

Despite the expectation of low levels of postnatal depression, epidemiological studies from sub-Saharan Africa (SSA) have reported prevalance estimates for postnatal depression which are comparable to those from high income countries (Aderibigbe, Gureje, & Omigbodun, 1993; Adewuya, Fatoye, Ola, Ijaodola, & Ibigbami, 2005; Cooper et al., 1999; Cox, 1983; Nakku, Nakasi, & Mirembe, 2006; Nhiwatiwa, Patel, & Acuda, 1998). As the majority of studies were conducted in urban centres, it is possible that societal transition has led to erosion of sociocultural perinatal practices and a resultant increase in postnatal depression. Alternatively, the high prevalence of postnatal depression could be accounted for by difficulties in the cross-cultural measurement of depression. Application of non-validated diagnostic concepts of postnatal depression could result in a ‘category fallacy’ (Kleinman, 1987), that is detecting pathology which is not recognised as such within the culture. A further possibility is that certain traditional sociocultural perinatal practices could have detrimental effects upon mental health.

### Postnatal depression as category fallacy

Few of the above epidemiological studies from SSA have grappled with the issue of cross-cultural measurement of postnatal depression, tending to define cases according to international diagnostic criteria (World Health Organisation, 1992). Our recent study found the widely-used Edinburgh Postnatal Depression Scale (Cox, Holden, & Sagovsky, 1987) to be culturally invalid in rural Ethiopia, although another measure demonstrated reasonable ability to detect cases of postnatal mental disorder as defined by Ethiopian psychiatrists (Hanlon et al., 2008). It remains the case that very few anthropological studies from SSA have systematically evaluated the presence, or absence, of postnatal distress states as recognised from within the specific culture (Harkness, 1987; Oates et al., 2004).

### Postnatal practices as potentially harmful to mental health

The relationship between traditional practices and postnatal mental health has not been systematically evaluated in SSA, although studies have identified traditional perinatal beliefs that might be detrimental to postnatal mental health (Chalmers, 1990; Cox, 1979). In non-Western countries, non-performance of postnatal traditions has been inconsistently associated with postnatal depression (Fisher, Morrow, Ngoc, & Anh, 2004; Lee, Yip, Leung, & Chung, 2004; Rahman, Iqbal, & Harrington, 2003) with evidence that performance of some postnatal traditions is associated with poorer mental health (Chan, Levy, Chung, & Lee, 2002; Grace, Lee, Ballard, & Herbert, 2001).

Given the dearth of systematic studies, particularly from SSA, we conducted a qualitative study in a predominantly rural area of Ethiopia. Our aim was to generate hypotheses that could subsequently be tested in a planned population-based cohort study of the epidemiology of postnatal common mental disorders. Postnatal mental disorders are customarily divided into maternity blues, postnatal depression and puerperal psychosis, although anxiety states and other disorders also occur (Brockington, 2004). As an exploratory study, we were interested in any distress states considered important from the perspective of the community. Distress states were defined to include both spiritual and mental disturbance because of the close association previously observed in Ethiopia (Alem, Jacobsson, Araya, Kebede, & Kullgren, 1999). Our research questions were as follows:

▪ Is there societal recognition of mental distress or spiritual problems specific to the postnatal period? Furthermore, does the community recognise postnatal mental disorders as conceptualised within international diagnostic frameworks?
▪ How does the occurrence of any identified mental or spiritual problems relate to sociocultural patterning of the postnatal period?

## Methods

### Study setting

The study took place in 2004 in and around the town of Butajira, located 130 km south of Addis Ababa. The Butajira Rural Health Programme (BRHP) was established in this area in 1985 to support public health research (Berhane et al., 1999). The Butajira area covers both urban and rural areas, as well as tropical lowland areas (altitude around 1500 m) and temperate mountainous areas (altitude up to 3500 m). Around Butajira, the livelihood of the residents is based on mixed farming, with Khat (an amphetamine-like psychostimulant) and chilli pepper as the main cash crops and maize as the main subsistence grain. With population expansion, this historically fertile area has become prone to food insecurity, affected by famine in 1974, 1985, 1999 and 2003. Childbirth remains hazardous for women in Butajira, with maternal mortality rates estimated at 400–850/100,000 births (Berhane, Andersson, Wall, Byass, & Hogberg, 2000). Fewer than 10% of women attend for delivery in a health facility or receive any formal postnatal care (CSA, 2006).

### Sample

We initially identified a key informant woman who had lived in the area all her life and worked for the BRHP for some years. She was able to make use of her good standing and diverse contacts in the various sub-districts to identify potential participants and to introduce us into the community. We wished to elicit a broad range of perspectives on pregnancy, birth and the postnatal period and purposively sampled 25 people for in-depth interview and 53 to participate in focus group discussions (FGD). We conducted in-depth interviews with pregnant (*n* = 2) and postnatal (*n* = 4) women, a community leader, an Orthodox Christian priest, a Muslim traditional healer (*kalicha*), leaders of women's religious groups (Christian and Muslim; *n* = 3), the gender officer from a local women's advocacy organisation, the head of Women's Affairs in the local government, two members of a women's micro-finance organisation, trained and untrained traditional birth attendants (*n* = 2), a midwife, a primary healthcare worker and two BRHP workers. Through snowballing (Bernard, 2006), we also followed up descriptions of women with postnatal distress states and interviewed three of these potential ‘cases’.

Five FGDs were conducted with the following groups: (i) postnatal women, (ii) pregnant women, (iii) traditional birth attendants (TBAs) with experience ranging from 2 to 30 years, (iv) grandmothers, and (v) fathers.

The sociodemographic characteristics of participants are summarised in Table 1.

### Protocol

All in-depth interviews and FGDs were conducted in Amharic, the official language of Ethiopia, by an Ethiopian midwife (AH) with previous experience in qualitative data collection. The majority were attended by the first author, an English psychiatrist who was living in Butajira. We introduced ourselves as ‘university researchers interested in learning more about women's health during pregnancy, birth and the postnatal period, the problems they encounter and their experiences’. Interviews were taped, transcribed in Amharic and translated into English prior to coding. For the majority of transcripts, CH typed in English as AH translated from Amharic. Any ambiguities or points of interest were discussed and noted within the transcripts. Four in-depth interviews and four of the FGDs were directly translated into English, with later discussion. They were conducted in a range of settings, including primary healthcare facilities, the BRHP project office, other office facilities or people's own homes. At all times privacy was assured. Non-professional participants received remuneration for transport costs.

The FGDs followed recommended methodology (Krueger & Casey, 2009). Two of the FGDs were conducted by AH. The FGDs with pregnant and postnatal women were conducted by female Ethiopian doctors. The FGD of fathers was conducted by a male nurse working on his PhD in the Butajira area and trained in qualitative methods. All FGDs were tape-recorded and a note-taker was present to facilitate later transcription, as well as to observe and document non-verbal communication. All facilitators were well-versed in the purposes of the project and demonstrated considerable aptitude in developing rapport with the participants and managing the group discussions.

### Topic guide

Interviews and FGDs were loosely structured around pregnancy, birth and the postnatal period. For the purposes of this paper, we will focus on the topics related to the postnatal period. Open questions were used to find out about the kinds of traditions and restrictions a postnatal woman might be subject to, the types of difficulties postnatal women can face and the sources of support they could expect to rely upon. Planned probes included direct questionning about previously-identified common attributions for mental illness in Ethiopia (Alem et al., 1999), namely evil eye, bewitchment, ancestral troubles, spiritual problems and mental (‘nervous’) problems in the postnatal period, with follow up questions aiming to elicit specific examples known to the respondent. The interviewer took care to be sensitive to topics initiated by the participant and to allow the interview to proceed in as naturalistic a manner as possible. The topic guide developed iteratively as the study progressed. At the end of the interview, two vignettes of women with postnatal depression, modelled on ICD-10 criteria, were read out and participants were asked whether they recognised such a problem in their area, and to comment on possible causes, course and outcome.

### Analysis

Initial analysis proceeded in tandem with data collection, with discussion of emerging themes between CH and AH. This facilitated iterative development of the topic guide and enabled us to triangulate data obtained from interviews and FGDs. To improve rigour of analysis, following completion of data collection, CH and DW independently coded four transcripts using descriptive codes. Coding schemes were compared and rationalised, with discussion about points of disagreement. CH and DW then applied these codes to two further transcripts as a cross-check. CH recoded the remaining transcripts, drawing upon additional codes where the data required. *Atlas.ti* computer software was used to facilitate data management (Muhr, 1997). In accordance with the tenets of inductive analysis (Glaser & Strauss, 1967), care was taken to allow codes to emerge from the data. Higher order themes were formed which were tested back against the data and discussed. Illustrative quotes were selected with agreement from all authors.

Ethical approval was obtained from Research Ethics Committees of the Ethiopian Science and Technology Agency and the Institute of Psychiatry, King's College London.

## Findings

Once the dangers of pregnancy and childbirth had been safely negotiated, the postnatal period was described, for the majority of women, as a time to be savoured. All participants recognised a demarcated period of postnatal confinement, ranging from 40 days up to 3 months. Participants identified rest and recovery, recognition of the mother's new status, spending time with the new baby and managing threats to both mother and baby, as the shared sociocultural patterning of the postnatal period. In relation to this postnatal structuring, participants identified situations which might be associated with abnormal distress states in the postnatal woman. These clustered around themes of ‘disappointed expectations and exclusion’, ‘exacerbating pre-existing problems’ and ‘vulnerability and danger’. All themes arose in all the groups of participants, whether they were educated or uneducated, male or female, living in an urban or rural residence, speaking as part of a group or in an individual interview.

### Theme I: disappointed expectations and exclusion

The cultural ideal for patterning of the postnatal period was strongly endorsed and pervasive across participants. This was accompanied by tacit acknowledgement that the actual experience of many women would fall short, leading to disappointed expectations and potential exclusion from social recognition. Such women were considered at risk of distress and at heightened vulnerability to spirit attack.

Rest was considered a necessity following the demands of pregnancy and childbirth in order to bring the new mother back to full health and functioning. The postnatal woman would expect to be provided with good food and cared for by her family, friends and neighbours. Whereas pregnancy was associated with secrecy, the postnatal time was spoken of as a time of celebration and social recognition. The moment that the woman gave birth to a healthy baby, the accompanying women would ululate in celebration. For the ceremonial burial of the placenta and, later, after being washed with water and herbs, relatives and women of the neighbourhood would join the postnatal woman to drink coffee and eat special porridge. The social obligation to visit the postnatal woman would ensure that she was congratulated by many and might receive gifts. If the family were able, the husband would slaughter an animal as part of a celebratory feast.

Participants identified a variety of ways in which this social recognition of the postnatal woman might be threatened, leading to mental distress or spiritual disturbance. Some women would be excluded by virtue of the circumstances of their pregnancy, for example, occurring outside of marriage. For others, celebratory visits and gifts would be contingent on whether the woman had fulfilled social duties towards postnatal women in the past. If the woman had been unable to prepare during pregnancy to entertain people in her home, most often through lack of resources, participants described sadness arising from the resulting shame and alienation. Most often, however, it was deficits in the care and recognition received from the woman's husband which were regarded as potent causes of disappointment and distress for the postnatal woman. As described by participants, the postnatal woman may be particularly vulnerable to emotional harm if the husband spends all his time with another wife, or, indeed, starts to see another woman.

Yes, there are women who are sad. Maybe if she doesn't have a good standard of living or if the husband doesn't care for her properly. If he doesn't do what is expected of him then the woman will be unhappy. Sometimes they even say ‘why did I have this baby? Rather than having this baby, it would have been better if I had died.’… Male rural community leader (IV24).

R8: Sometimes if the postnatal time is not like the previous one, this also can hurt her feelings and she might have an emotional problem…

R1: …there are people who have two wives. If one delivers, the man will stay with the other wife. So this might also create a problem because the other woman feels she is being neglected. So she might have this emotional problem. The fathers FGD.

As well as emotional harm, disappointment arising from the husband's behaviour was recognised by participants as increasing vulnerability to spirit attack (‘*likift*’), manifested by signs of mental and behavioural disturbance:

…She might have a sudden illness. People call this thing ‘*Likift*’… She will be disturbed and become irritable. She will disagree with her husband about everything. For me, this kind of thing could be due to a decrease in the love and affection he had before and she could become unhappy and like this… This is also the time when she needs more care from the husband. So even though it is small, if he changes a bit, she will notice and it will be a big thing for her. She will worry about it and become unhappy. Leader of urban women's micro-finance group (IV19).

It was clear that ‘*likift*’ did not always give rise to mental symptoms and was not an affliction specific to the postnatal period, although postnatal were considered to be spiritually vulnerable.

Participants observed that postnatal celebrations would be greater following birth of a boy. When asked whether a boy or girl baby was preferred, almost all replied that the husband would prefer a boy even if the woman might want to have a girl. Boys were said to confer higher status upon the woman, as well as inheriting the family's property and wealth. Having repeated girl babies was seen as diminishing a woman's status and a threat to her happiness in the postnatal period.

Boys are preferred. I myself was expecting it [my baby] to be a boy and when I saw the baby, I said ‘She must be a boy’. The traditional birth attendant said ‘She is a boy’. But when the baby cried, I knew that she was a girl and I was disappointed. Urban postnatal woman (IV06).

### Theme II: exacerbating pre-existing problems

Distinct from distress arising from an inability to adhere to the postnatal traditions, participants also recognised women for whom the postnatal period of confinement exacerbated their pre-existing difficulties. Running through this theme was a concern with the woman's diminished autonomy during the postnatal period that served to undermine her usual survival strategies. The restrictions in her ability to share her problems with others, to make her own money or escape from the home might render the ever-present threats from poverty, marital discord and ill-health overwhelming.

The power of the tradition of postnatal confinement meant that many women felt unable to leave the postnatal house for fear of being shamed or inviting other adverse consequences. At its worst, postnatal confinement could serve to hide suffering from the public eye:

If she doesn't have somebody near her to support her, to prepare her food and drink, it is a tragedy… Everybody thinks that she has everything, and her husband will act as if he is giving her everything. He may punish her with hunger and nobody knows. She wouldn't go out to seek help because she is afraid of the tradition and that she may get hurt… If she goes out before two months have passed she is either selfish or shameless. Women's affairs officer (IV22).

The marital relationship was noted by participants to be strained by the additional demands of the confined woman and the new baby, particularly when the household was struggling at the limits of survival. For many families, the woman's confinement would mean a loss of income as she would be unable to engage in small-scale trading and other income-generating activities. Moreover, confinement could represent a loss of financial autonomy for the woman. Unless she had been able to save beforehand, without her independent income, however, meagre, the woman would be fully dependent upon her husband to provide for her, the new baby and her other children. The resulting necessity to make requests of her husband was seen as a source of arguments, abuse and insecurity. A potent source of worry for the postnatal woman was that the baby might become ill or die. Such fears were heightened by the need to depend on her husband for money and permission to seek healthcare should the baby fall sick. Women already living in abusive marriages might be at greater risk during the postnatal period. The impact upon the woman's mental well-being was described by one woman with firsthand experience of being beaten by her husband:

I never had a happy day during that postnatal period… It was my living [circumstances]. The problem in my house was because my husband and I quarrelled at that time and he didn't help me. He didn't even come to my house to visit me…. During that time I felt hopeless most of the time…. I thought about everything. I even thought about ending my life or leaving everything and going somewhere else…. Yes. At that time, had I been God or had I been the person who can do anything, I thought of killing her [her baby] and killing myself…. Since I didn't have the guts to kill the baby or kill myself, I just thought about it. Rural postnatal woman (IV15).

For this woman, her postnatal status meant that she was unable to escape and so she had to stay and endure. For postnatal women in general, participants indicated that a major source of distress was being unable to share their problems with friends and neighbours.

Yes, there is definitely a problem with worry. Because she is not allowed to go to other people's houses, talk with them and share what she would like to share. Because this will give you pleasure and happiness. But if a woman is in the postnatal period, she has to stay at home. When she is in the house, she will be alone. So if she doesn't have anyone to talk to or someone near to her to send out for things she needs, this might change her mood and she will create arguments with others… Fathers FGD.

Postnatal confinement in vulnerable women was associated with spontaneous descriptions of a range of symptoms of mental distress, including sadness, irritability, social withdrawal, suicidal ideation, hopelessness and even thoughts of harming their baby. Almost all these symptoms were attributed to marital problems, with or without the further stress of poverty, and, for most, the solution proffered was to go back to their family, to share their problems with friends or for the community elders to assist in settling any dispute.

Female participants who were involved in urban institutions (micro-finance groups, local government and advocacy groups) were more likely than their rural counterparts to speak of problems in the marital relationship. Almost all the male participants openly debated domestic violence and the problems associated with women's low autonomy.

### Theme III: vulnerability and danger

For some women, participants identified threats to their well-being, including their mental and spiritual health, arising from the postnatal woman's inherent vulnerability and the consequences of not following prescribed practices. After safely navigating the dangers of pregnancy and childbirth, the postnatal woman and her newborn baby are considered to become prey to a different set of threats: spirit attack and possession, evil eye, bewitchment and exposure to harmful draughts of air (*nefas)* and sunrays (*mitch)*. Illness and death may result. Countering these threats to the postnatal woman and her baby, and the concomitant threat to society at large, entailed tight restrictions on how she should behave. The postnatal woman's food and sleeping area would be separated from the rest of the family in the first days after birth, with no gaps where draughts of air or sunlight might penetrate through. For the duration of the postnatal period, she would be restricted from crossing the perimeter of her compound or participating in usual social obligations. She should not leave the darkness and safety of the postnatal home when the sun was high overhead and she should carry metal, garlic and herbs with her on any forays out of the house. Her body demeanour should convey to all that she was in the postnatal house, required to walk slowly and speak softly. She should cover up her baby and use traditional medicines and charms to protect him or her from those who might have malicious intent and she should not incite envy by using excessive butter on her hair. In itself, keeping the traditions was seen as heightening the woman's level of anxiety:

Let me tell you something. In our tradition, for example, women in the postnatal period have to decrease even the amount that she talks. She is not allowed to talk in a loud voice. So if she talks louder, they will tell her to decrease her voice ‘Don't talk loudly’. So this might upset her or might make her worry. Fathers FGD

Fear of violating postnatal rituals was also a concern, with participants relating numerous stories of postnatal ill-health and misfortune attributed to inadequate adherence to the requisite protective practices.

Others say she might have this *Likift* when she goes outside the postnatal house where she might be exposed to *Djinne* [a spirit]. In our area they call it *Likift*. Some of the women with this kind of problem, sometimes they even want to kill their baby or they may refuse to breast-feed their baby. Rural postnatal woman (13).

Participants frequently described supernatural agents, such as *likift or djinne*, causing disturbance of the postnatal woman's thinking or behaviour, for example thoughts of harming her baby, aggression, talking to herself or speaking nonsensically. Although these symptoms formed only part of a wide range of possible, often overlapping, manifestations, they tended to be more suggestive of severe mental disorder when compared to distress states attributed to disappointment, poverty and marital difficulties.

The spiritual dangers of the postnatal period are reinforced by the existence of a special group of women within Butajira society who are afflicted by spirit possession, so-called *wuqabi*. Two of the women interviewed for the study identified themselves as suffering from *wuqabi*. Other participants were mostly aware of such women in their midst, although at times appeared notably uncomfortable discussing them. As described by our participants, both women and men can be possessed by these spirits, requiring them to worship the spirit and perform certain rituals. Usually the spirit is passed down the generations within a family. Although the spirit requires attention at all times, the postnatal woman is particularly vulnerable to disturbance because of the blood associated with childbirth and the puerperium. As one of the women affected by *wuqabi* describes:

I was not happy in the first few days of my postnatal period. It was not pleasant. As for any postnatal woman I was kept in the dark behind the curtain and I didn't like it. I got some relief when I pulled up the curtains and got some ligh… I got sick from the moment I give birth… I couldn't act like a postnatal woman. There are things you are expected to do in the postnatal period. For example, they put butter on my head. I would throw it away. I wanted to be clean. Otherwise I don't feel healthy. I don't even like to visit postnatal women… After I have visited a postnatal woman I get a headache for some time, my face burns and I get nervous. Rural woman with *Wuqabi* (IV29).

For the postnatal women with *wuqabi*, it is the expectations and constraints of postnatal practices which make the ancestral spirit unhappy and cause the woman to report ‘disturbance’. For such a woman, violating the traditions is the cure for her ills rather than the cause.

The male participants spoke most freely about perceived supernatural aetiology to postnatal problems, whereas rural, older women with little education showed some reluctance to discuss this topic in any depth. Although urban, female participants tended to say that they did not fully subscribe to these beliefs, they supported the notion that such concerns were widespread.

### Recognising postnatal depression: vignettes

(1) A woman from my home area gave birth 2 months ago. She is now always arguing with her husband and says she wishes she had never had her baby. She thinks that nobody helps her and that she can't manage on her own. She doesn't know what to do. Sometimes she just cries.
(2) Another woman who had a baby recently. She used to be friendly, but since giving birth has become very withdrawn. She doesn't seem to notice things around her. She even doesn't remember to feed her baby or herself. Sometimes she says it would be better for everybody if she was dead.

For both vignettes, around half of participants said they had never seen such a woman. Many participants seemed uncomfortable, particularly with the second vignette and the expression of suicidal ideation, and seemed keen to quickly change the subject. Some participants did, however, recognise the vignettes and elaborated their ideas as to what could be the cause of such a malady. The attributions largely accorded with the identified themes of threats to mental health. Of the two women with *wuqabi*, one identified herself with vignette (1), the other with vignette (2). One respondent, who had spontaneously described experiencing a number of symptoms suggestive of depressive disorder, recognised the symptoms but resisted pathologisation of her experience:

R: …[pauses]…it seems that it is the kind of problem I had in the postnatal period.

I: Did you have these kinds of problem in the postnatal period?

R: Yes, I told you about it. It seems like this but I don't think it was a kind of illness, I just had the problems. Rural postnatal woman (IV15).

Most commonly, vignette (1) was attributed to marital problems, insufficient care from her husband and disappointment. Other possible causes were mentioned, including an unwanted pregnancy or birth outside of wedlock, poverty and hunger, violation of a postnatal taboo and the influence of a supernatural agent (spirit possession, *wuqabi* or evil eye).

R4: Yes, there are women who will say ‘why did I give birth?’ if they don't have the necessary care or support from their husband, or from the rest of the family. Or if she doesn't have enough food to eat. Even apart from having enough food, if she doesn't have the happy face of the husband, she might become unhappy or cry.

…

R10: There are women who aren't happy right after delivery. There are women who cry, not only cry but sometimes they want to die. Pregnant women FGD.

Vignette (2) was more uniformly attributed to supernatural causes, particularly spirit possession, although often participants seemed uncertain. A few participants also implicated unwanted pregnancy, disappointment, violation of postnatal taboos and marital discord as contributing to the woman's difficulties.

## Discussion

With respect to our original objectives, we found that, although the postnatal period was highly socioculturally elaborated and considered to be a good time in a woman's life, participants from this rural area of Ethiopia did identify problematic distress states in postnatal women. Vignettes of postnatal depression according to international diagnostic criteria were recognised but not considered as illness. Abnormal distress was noted in relation to cultural patterning of the postnatal period intertwined with broader societal power relationships and economic strain.

Participants spoke of the difficulty facing women who are unable to participate in postnatal practices which they consider important or who otherwise missed out on receiving full recognition as a new mother (‘disappointed expectations and exclusion’). The constraints of the postnatal period were seen as problematic for some women when interacting with gender disadvantage and poverty (‘exacerbating pre-existing problems’). The inherent dangers of the postnatal time, and the supernatural threat posed by non-adherence to postnatal rituals, seemed to be associated with more severe manifestations of mental disorder (‘vulnerability and danger’).

### Disappointed expectations: the role of cultural dissonance

Participants identified the mismatch between expectation and actuality as a potent stressor for some postnatal women, leading to manifestations of mental distress. At one level, this finding supports Stern and Kruckman's contention that postnatal practices may be protective against development of mental distress. Even in cultures where the postnatal period is extensively elaborated, as in Butajira, some women may miss out on the purported benefits of traditions, such as provision of social support and social recognition, placing them at heightened risk of mental ill-health. However, rather than absence of postnatal traditions being the problem as is hypothesised for Western women, in Butajira it is the strength of the prevailing sociocultural norms surrounding the postnatal period which emphasises the social exclusion of non-participating women and leads to distress. This resonates with Dressler's concept of ‘cultural consonance’ whereby the degree to which individuals, in their own beliefs and behaviours, approximate widely shared cultural models, influences health outcomes, including psychological well-being (Dressler, Baleiro, Ribeiro, & dos Santos, 2007a and 2007b). When the individual is unable to adhere to strongly held culturally values, for whatever reason, cultural dissonance results and can lead to depression (Dressler, Balieiro, Ribeiro, & dos Santos, 2007b). The greater the consensus in a cultural domain, the more strongly cultural dissonance will be associated with development of depression (Dressler et al., 2007b). Therefore, it is the very mismatch between internalised cultural expectation and actual behaviour which increases risk of mental distress.

Societal transition and worsening of the economic situation may be eroding women's capacity to adhere to perinatal rituals, as evidenced by a recent study from Butajira showing that the younger generation of women were less likely to have completed even 1 month of postnatal confinement compared to the experience of the older generation (59.3% vs. 68.8%) (Berhane, Gossaye, Emmelin, & Hogberg, 2001). In this climate, cultural dissonance may be on the increase and would be a testable risk factor for development of postnatal depression in this setting.

Societal gender preference for the newborn baby emerged as a domain imbued with much cultural expectation and, therefore, ripe for emergence of cultural dissonance. Preference for a male baby was strongly endorsed by the participants in this study, particularly when reporting the perceived view of the husband. In Ethiopia, unlike countries of South Asia (Sen, 2001), there is no imbalance in the ratio of men to women or suggestion of ‘missing women’ due to selective abortion or differential care (Population Census Commission, 2008). However, favouring of boy children has been noted in terms of nutritional status (Koohi-Kamali, 2008) and access to education (Berhane et al., 2001). An association between ‘gender bind’, that is giving birth to a girl where the birth of a boy is strongly culturally desired, and postnatal depression has been found in South Asia (Patel, Rodrigues, & DeSouza, 2002). Studies from sub-Saharan Africa have yielded mixed results, likely reflecting the tremendous heterogeneity of cultures across the continent (Aderibigbe et al., 1993; Adewuya et al., 2005; Cooper et al., 1999). The relationship between gender preference and postnatal depression has yet to be empirically tested in Butajira, Ethiopia, but our results suggest an association worth investigating.

### Exacerbating pre-existing problems: poverty and gender disadvantage

An unexpected finding from informant reports was that the restrictions of the postnatal period could be associated with increased distress if the postnatal woman already had difficulties, particularly related to poverty and gender disadvantage.

Extreme poverty has been associated with depressive illness in non-perinatal Butajira women (Deyessa, Berhane, Alem, Hogberg, & Kullgren, 2008) and poorer perinatal outcome (Byass, Fantahun, Mekonnen, Emmelin, & Berhane, 2008). As described by study participants, chronic poverty interferes with women's ability to engage in acts of reciprocity and blocks off avenues of social support. Economic difficulties, coupled with the societal change alluded to above, may, therefore, pose the biggest challenge to women's mental health regardless of whether or not she is in the perinatal period. Our data do, however, suggest that the strains of poverty become more acute in the puerperium as the woman is temporarily excluded from the cash economy at the same time as having an extra mouth to feed.

Other recent studies from the Butajira, Ethiopia, have drawn attention to the disadvantaged position of women within this transitional society (Berhane et al., 2001; Gossaye et al., 2003). Berhane argues that women's status and living conditions may be particularly vulnerable because of longstanding gender disadvantage amongst rural Ethiopian women, including low levels of education and literacy (CSA, 2006), lack of autonomy, domestic violence and polygamy, all of which may restrict a woman's coping options (Berhane et al., 2001). In our study, polygamy was considered a source of discontent amongst postnatal women. The time when a man takes another wife has previously been recognised as a potent threat to a women's psychological well-being in Butajira (Berhane et al., 2001), with culturally prescribed remedies to elevate the status of the original wife.

Poor marital relations in general were seen by almost all participants as a great problem for the confined postnatal women. In a WHO-sponsored multi-country study of intimate partner violence, Butajira, Ethiopia had a high 12-month prevalence of physical (29%) and sexual violence (44%)(Garcia-Moreno, Jansen, Ellsberg, Heise, & Watts, 2006), both of which were associated with depressive and anxiety disorders (Deyessa et al., 2009). In the Ethiopia sample, physical violence was increased in pregnant women (Berhane et al., 2001). Nearly one-third of Butajira women experiencing intimate partner violence coped by escaping from the home. The decreased mobility of pregnant and postnatal women may, therefore, foreclose the possibility of an important coping strategy and worsen their distress.

### Vulnerability and danger

Vulnerability of the postnatal mother and her newborn child to supernatural attack was a prominent theme, underpinning many of the protective rituals following birth. From the participants' accounts, it was evident that the very performance of these protective rituals served to reinforce underlying fears and increase anxiety levels in susceptible women. When participants described severe illness, or even death, occurring in the postnatal period this was often attributed to violation of postnatal taboos or neglect of prescribed practices exposing the woman or child to risk of spirit attack. The descriptions of illness attributed to supernatural causes did not describe a particular syndrome, although when symptoms of mental disorder were present they tended to indicate severe disturbance such as might be found in cases of puerperal psychosis. This is in keeping with previous Ethiopian studies examining illness attribution in non-perinatal mental disorder which found symptoms indicative of psychotic states to be more often attributed to supernatural cause (Alem et al., 1999), whereas depression and anxiety were more likely to be linked to socioeconomic conditions and interpersonal difficulties (Deribew & Tamirat, 2005; Mulatu, 1999). Caution needs to be exercised in mapping the descriptions of;our participants onto international diagnostic categories. In Butajira, a previous study showed something of a mismatch between cases of psychotic disorder identified by community participants compared to clinical interview (Shibre et al., 2002). Furthermore, expressions of spiritual or mental disturbance have a symbolic power in and of themselves which may not be reducible to Western conceptualisations of intrapsychic pathology (Boddy, 1988).

The culturally deviant case of *wuqabi*, reinforces the power of the postnatal restrictions, equating violation with affliction by ancestral spirit possession. In such a climate of danger, deliberate or inadvertent lapses in adherence to postnatal rituals could be threats to mental health in some women.

### Recognition of postnatal depression

Respondents spontaneously described problematic distress states manifested by sadness, irritability, hopelessness and suicidal ideation, which are in keeping with the Western construct of postnatal depression. However, participants did not identify a specific illness entity afflicting the mental health of mothers in the postnatal period. Vignettes of Western-defined postnatal depression were not recognised by a large proportion of participants. Furthermore, although the vignettes elicited similar attributions to those described spontaneously as problematic distress states, these were not considered to be illness. This is in keeping with previous Ethiopian studies that found low recognition of Western constructs of depressive illness compared to other mental illnesses (Alem et al., 1999). Although depressive symptoms were recognised, as with previous studies, the disturbance was was explained in terms of social adversity, with individualistic explanations for illness uncommon (Deribew & Tamirat, 2005; Mulatu, 1999). Our findings are also in keeping with a cross-cultural qualitative study exploring abnormal postnatal distress in 11 countries, including Uganda. Such distress states were recognised across all settings although, in Uganda, were not considered to constitute mental illness (Oates et al., 2004).

Kirmayer warns that, in examining cultural differences in distress, we are constrained by our biopsychosocial model which allows for distress to be expressed in somatic, psychological and sociomoral idioms. Beyond this, we may neglect religious, supernatural or cosmological idioms which fall outside the reified categories of science (Kirmayer, 1989). In this regard, it is interesting that the two women with enduring spirit possession (*wuqabi*) both identified themselves with the Western vignettes and were recognised by other participants as having a culturally undesirable affliction or illness. A similar form of spirit possession, *Zar*, has been well-studied within Ethiopia and neighbouring countries, although not in relation to the perinatal period (Fuller Torrey, 1967). The condition has been said to be adaptive, enhancing the status of the afflicted person and giving a voice to marginalised and subordinate members of society (Messing, 1958; Young, 1975). In our study, the woman with *wuqabi* was somewhat feared. She was allowed relative freedom to act against postnatal norms of behaviour and her condition entitled her to make demands of others which would otherwise not be fulfilled. Despite these apparent advantages, both affected women and other participants considered *wuqabi* to be a highly undesirable affliction. Concern about personalised supernatural attack, for example evil eye or bewitchment, was not as prominent as concerns with spiritual possession or encounters with evil spirits.

### Limitations

Ethiopia has a diverse population, with over 80 ethnic groups (Central Statistical Authority (CSA), 2008), potentially limiting the generalisability of our findings. Furthermore, the BRHP area is predominantly Muslim, whereas Ethiopia as a whole has a majority Christian population. Although such variation may have affected the specific details of perinatal traditions, our main themes may well be applicable to the majority of rural Ethiopian women, given the widespread experiences of poverty and gender disadvantage. Our study was also limited by the focus on negative emotions. As the predictors of perinatal happiness and unhappiness are not necessarily the inverse of one another (Oates et al., 2004), we may have missed out on a full appreciation of factors affecting well-being of the perinatal woman.

### Postnatal practices and postnatal mental distress: hypothesis-generating

In common with other non-Western settings, the postnatal period in rural Ethiopia is extensively culturally elaborated and yet participants did identify distress states in postnatal women. Although not considered as illness, these distress states were considered to be problematic. The relationship of these distress states to the postnatal mental disorders identified by international diagnostic criteria needs further exploration. Women at risk of developing such distress states might be those whose postnatal experience is dissonant with internalised cultural expectations, including inability to participate fully in the postnatal period or giving birth to a girl baby. The potential role of poverty and gender disadvantage as effect modifiers for the impact of postnatal traditions upon maternal mental distress could be quantitatively investigated. Non-adherence to protective rituals may exacerbate fears and insecurities and risk of spiritual disturbance. The mental health status of women with *wuqabi* deserves further examination.

## Acknowledgements

We gratefully thank all participants for contributing their time and energy to the study. In addition, we wish to extend our thanks to all those at the Butajira Rural Health Programme who facilitated this project AND AYNALEM HAILE-MICHAEL FOR CONDUCTING THE INTERVIEWS. The Wellcome Trust is gratefully acknowledged for funding the study through a Research Training. Fellowship for Dr. Charlotte Hanlon.

## Figures and Tables

**Table 1:** Sociodemographic characteristics of respondents.

	In-depth interviews	FGD 1 Postnatal women	FGD 2 TBAs	FGD 3 Grandmothers	FGD 4 Fathers	FGD 5 Pregnant women
Number of participants	25	11	11	10	10	11
Duration (hours)	0.75–2.00	1.50	3.00	2.50	2.50	2.00
Age (years)	21–70	16–31	20–60	40–75	21–39	19–33
Gender (female)	19	11	11	10	0	11
Rural or urban residence	15 urban, 10 rural	All rural	Majority rural	All rural	All rural	All urban

Marital status						
Single	3	–	1	–	–	–
Monogamous	13	7	9	3	10	8
Polygamous	5	4	–	2	0	3
Separated	2	–	–	–	–	–
Widowed	2	–	1	5	–	–

Schooling						
None	12	10	8	9	–	6
Grade 1–9	6	1	3	1	10	5
≥ Grade 10	7	–	–	–	–	–

Religion						
Muslim	13	11	7	8	7	9
Orthodox	10	–	3	2	2	2
Protestant	2	–	1	–	1	–

Ethnicity						
Meskan	13	–	6	9	8	11
Mareko	1	–	1	–	–	–
Silti	2	11	1	–	–	–
Other	9	–	3	1	2	–
